# Shear-wave ultrasound elastography for tissue-specific quantification of radiation-induced cervical fibrosis

**DOI:** 10.1016/j.phro.2026.101005

**Published:** 2026-05-27

**Authors:** Hendrik Dapper, Maria Waltenberger, Steffi U. Pigorsch, Stephanie E. Combs, Katharina Bauermeister, Wolfgang Bauermeister

**Affiliations:** a Department of Radio-Oncology, Klinikum rechts der Isar, Technical University of Munich, Ismaninger Str. 22, 81675 Munich, Germany; b Department of Radiation Oncology, Cyberknife and Radiation Therapy, Faculty of Medicine and University Hospital of Cologne, University of Cologne, Kerpener St. 62, 50937 Cologne, Germany; c German Cancer Consortium (DKTK), Partner Site Munich, a Partnership between DKFZ and University Hospital Klinikum Rechts der Isar, 81675 Munich, Germany; d Department of Radiation Sciences, Germany, Institute of Innovative Radiotherapy, Helmholtz Zentrum München, 85764 Oberschleißheim, Germany; e Department of Neurology, Klinikum rechts der Isar, Technical University of Munich, Ismaninger Str. 22, 81675 Munich, Germany; f Conservative and Rehabilitative Orthopaedics, TUM School of Medicine and Health, Technical University of Munich, Campus D, Am Olympiacampus 11, 80809 Munich, Germany; g Schmerzinstitut München, Associated Institute at Kharkiv National Medical University, Toni-Schmid-Str. 45, 81825 Munich, Germany

**Keywords:** Shear-wave ultrasoundelastography, Radiation, Fibrosis, Head and neck cancer

## Abstract

**Background and purpose:**

Radiation-induced cervical fibrosis is a common late effect following radiotherapy for head and neck cancer, potentially leading to reduced tissue compliance and functional impairment. Objective assessment, however, remains challenging in clinical practice. Therefore, this study aimed to evaluate the feasibility of shear-wave ultrasound elastography (SWE) for quantitative, tissue-specific assessment of radiation-induced cervical fibrosis.

**Materials and methods:**

Sixteen patients after bilateral cervical lymphatic irradiation were compared with sixteen age-, sex-, and body mass index–matched controls without prior head and neck irradiation. SWE was used to quantify stiffness of muscle, fascia, and subcutaneous tissue of the sternocleidomastoid muscle. Ultrasound-based thickness measurements and assessment of cervical range of motion were additionally performed.

**Results:**

Mean muscle stiffness was significantly higher in irradiated patients compared with controls (left: 46.98 vs. 28.10 kPa; right: 46.75 vs. 21.52 kPa). Comparable increases were observed in the fascia (left: 46.41 vs. 25.47 kPa; right: 44.28 vs. 22.69 kPa), whereas differences in subcutaneous tissue were less pronounced. Muscle and skin thickness were significantly reduced after radiotherapy. Cervical range of motion did not differ significantly between groups, although a non-significant reduction of approximately 5° in cervical rotation was observed. Negative correlations between muscle stiffness and cervical range of motion were observed but did not reach statistical significance.

**Conclusion:**

Shear-wave elastography enables reliable, tissue-specific quantification of radiation-induced cervical fibrosis, predominantly affecting muscle and fascia. Despite increased tissue stiffness and reduced thickness, cervical range of motion remained largely preserved, suggesting a dissociation between structural tissue alterations and functional impairment.

## Highlights

• Shear-wave elastography enables tissue-specific quantification of radiation-induced cervical fibrosis.
• Mean cervical muscle stiffness increased significantly from 21.5 kPa to 47.0 kPa after radiotherapy.
• Mean fascial stiffness increased from 22.7 kPa to 46.4 kPa after radiotherapy.
• SWE detects relevant tissue stiffening without significant impairment in cervical range of motion.

## Graphical abstract

**Figure:**
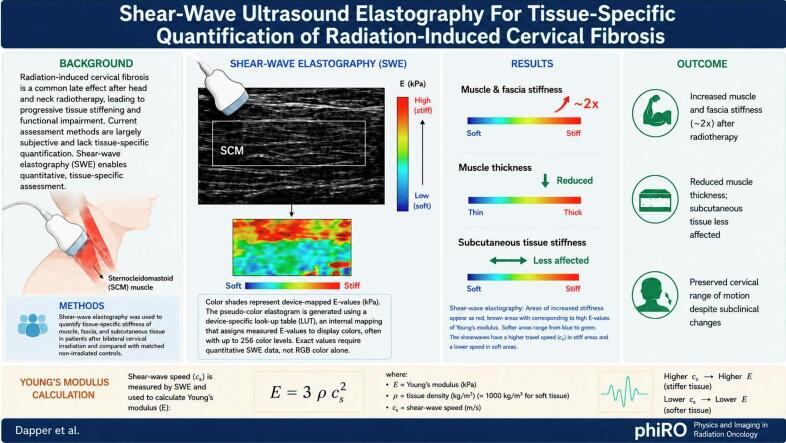

## 1 Introduction

Fibrosis is a descriptive term for a dynamic pathological process involving damage to parenchymal cells, stromal tissue remodeling, and contraction of the affected tissue [1]. Fibrosis is a late effect of radiotherapy that rarely improves over time and persists as a chronic condition impairing a patient’s functioning and quality of life (QoL) [2], [3], [4], [5]. Essentially, fibrosis results from a misdirected wound-healing process [5]. A crucial feature of fibrosis is hardening of the affected tissue. Blood and lymph vessels get displaced, and fat reservoirs are damaged, leading to edema, ischemia, and increased vulnerability to injury [1], [6], [7]. Consequently, the tissue atrophies and loses its functionality [1], [8]. Currently, there is no universally accepted definition of radiation-induced fibrosis. The pathophysiological changes and the resulting symptoms are referred to as”fibrosis”, depending on the context [1], [9]. Thus, the term is often used as an umbrella concept for chronic radiogenic changes in various tissues and organs and their resulting symptoms. It remains unclear which specific pathophysiological changes of diverse tissues cause which symptoms and functional deficits and how these in turn affect QoL. This illustrates the need for more specific assessment of tissue changes and their impact on clinically relevant outcomes. One group that is particularly affected by chronic soft tissue changes after radiotherapy is patients with ear, nose and throat (ENT) tumors. Due to the close anatomical relationship between various types of healthy tissue and the irradiated tumor, radiation-induced cervical fibrosis has particular importance in this group. The process impairs QoL through complications, such as cervical dystonia, muscle contracture, dysphagia, and neck muscle atrophy [9], [10], [11]. With modern intensity modulated radiotherapy [12], symptomatic fibrosis occurs less frequently compared to conventional 3D radiotherapy. However, approximately 30% of patients still develop grade 2 or higher symptomatic fibrosis one year after radiation [3].

Diagnostic methods for detecting radiation-induced cervical fibrosis are diverse [13], [14], [15], [16], because fibrosis involves complex, multifactorial pathophysiology and no diagnostic standard exists. Diagnosis generally involves detecting tissue hardening or imaging-based morphological changes such as induration. Historically, all methods have limitations. Manual palpation of radiation-induced stiffness in neck muscles can be scored, but is subjective [17]. Measuring Young’s modulus of elasticity in kPa through compression with a known force has been used to validate palpatory findings, with Young’s modulus correlating with palpation scores [18]. Indentometry, which measures stiffness in Newton per mm (N/mm), is another objective method [19]. However, both assess only overall stiffness without distinguishing whether subcutaneous tissue, fascia, or muscle contributes to it.

Shear-wave elastography (SWE), first described in 1995 by Sarvazyan et al. [20], is a quantitative method for stiffness measurement [14]. SWE is based on acoustic radiation force–induced shear waves propagating orthogonally to the excitation axis [21], [22]. The primary physical parameter measured is shear-wave velocity (Cs, m/s), which increases with tissue stiffness. Elastic moduli such as Young’s modulus (E, kPa) are derived parameters calculated from Cs under simplifying assumptions of tissue homogeneity and isotropy. Although stiffness values are commonly reported in kPa for clinical interpretability, velocity-based measurements more directly reflect tissue mechanical behavior, particularly in heterogeneous and anisotropic tissues such as muscle and fascia. The validity of SWE has been demonstrated in a histology-controlled mouse model of skeletal muscle fibrosis [23], and excellent inter-rater reliability has been reported for musculoskeletal SWE [24].

This study aimed to evaluate the feasibility of shear-wave ultrasound elastography for tissue-specific quantification of radiation-induced cervical fibrosis in patients treated with radiotherapy for ENT cancer. Furthermore, the study aimed to compare tissue stiffness of muscle, fascia, and subcutaneous tissue with a matched control group and to investigate associations between tissue stiffness and cervical range of motion (CROM).

## 2 Materials and Methods

### 2.1 Patient selection and functional assessment

The study was approved by the Ethics Committee of the Technical University of Munich (ethical approval number: 360/Sarva S-EB). From 2020 to 2022, 16 ENT cancer patients, predominantly with oropharyngeal carcinoma (n = 9), were selected as the observation group (OG). They had been treated between 2016 and 2021 with definitive radiochemotherapy, including bilateral regional lymphatic drainage pathways (at least level IIa to IVb). All patients received intensity-modulated radiotherapy with 50 Gy (2.0 Gy per fraction) to elective cervical lymphatic drainage (levels II–IV bilaterally) and a boost to 70 Gy for macroscopic disease, combined with 4–6 weekly cycles of cisplatin (40 mg/m^2^). For inclusion in the study, completion of oncological treatment had to be at least 16 weeks prior. The median interval from treatment completion to study inclusion was 26 months (range 6–60). All patients in the OG exhibited clinical mild cervical fibrosis. Exclusion criteria were operations or functional restrictions in the head and neck region involving the musculoskeletal system (such as chronic pain or restricted shoulder and neck movement) before radiation. Furthermore, no recurrence and no oncological treatment other than definitive radiochemotherapy had been performed or was pending. A control group (CG) of 16 patients was also included. They had not received any radiotherapy in the head and neck region or the shoulder, and had never undergone systemic therapy for oncological disease. Patients in the CG were matched to the OG by gender (12 men/4 women per group), age (median 65 vs. 66 years), and body mass index (20.3 kg/m^2^ vs. 22.7 kg/m^2^). Patient characteristics of the observational and control group are summarized in Table 1.

**Table 1:** Patient characteristics of observational and control group.

**Characteristic**	**OG (n = 16)**	**CG (n = 16)**
Age, years (median, range)	65 (52–76)	66 (54–77)
Sex, n (%)		
Male	12 (75%)	12 (75%)
Female	4 (25%)	4 (25%)
Body mass index, kg/m^2^ (median, range)	22.7 (18.0–27.5)	20.3 (17.7–25.7)
Time since radiotherapy in months (median, range)	26 (6–60)	–

OG = observation group, Med = median, CG = control group.

In addition, validated and widely used questionnaires were completed: the general oncological quality of life questionnaire EORTC QLQ-C30 and the head and neck-specific questionnaire EORTC QLQ-HN35. The thickness of the skin, subcutaneous tissue, and muscle was measured with an electronic caliper in offline mode using ultrasound. Furthermore, CROM was measured in both groups with the CROM Instrument (Performance Attainment Associates, Roseville, Minnesota, US) with a two-degree grading scale (Supplementary Material *A,* Fig. S1). A compass goniometer measured cervical rotation [25] (Supplementary Material *A,* Fig. S2) and a gravity goniometer measured cervical lateral flexion. The CROM device has shown good inter- and intra-rater reliability [26].

### 2.2 Ultrasound examination with shear-wave elastography

Shear-wave elastography (SWE) was performed using two-dimensional shear wave speed mapping on a Resona 7 ultrasound system (Mindray, China) with a linear *L*11-3U transducer (5.6–10.0 MHz), as previously described for musculoskeletal applications using this platform [27], [28], [29], [30]. Measurements were acquired using the manufacturer’s musculoskeletal elastography preset with High-Speed acquisition enabled (500 frames/s). Imaging depth was set to 4.0 cm, consistent with recommended depth ranges for reliable muscle SWE [27], [31].

Acoustic output parameters, including pulse intensity and effective transmit frequency, were automatically controlled by Qualitative General Penetration (QGen), with acoustic exposure continuously monitored via the Mechanical Index (MI), which remained within 1.0–1.2, in accordance with regulatory safety limits and SWE safety recommendations [31], [32].

A fixed rectangular region of interest (ROI) (30 × 25 mm) was positioned entirely within muscle tissue at depths of approximately 0.2–2.7 cm, avoiding fascia and artefacts. The shear-wave push pulse was generated at the ROI depth, resulting in a system-defined effective push depth within the typical operating range of linear transducers (∼1–4 cm) [31], [32]. The quantitative color-filling ratio was used as a system-derived quality metric reflecting spatial measurement reliability within the ROI and not as a direct measure of tissue stiffness. Depth-related color dropouts, predominantly observed at greater depths, were attributed to technical factors such as attenuation and reduced shear-wave tracking reliability rather than intrinsic tissue properties [31].

Only elastograms demonstrating homogeneous color filling and stable quantitative stiffness values, indicated by a five-star reliability index, were included in the analysis [28], [29], [30], [31], [33], [34]. Reported values represent the mean of eight consecutive acquisitions automatically averaged by the system. Measurements were repeated if within-measurement variability exceeded 15% or if the reliability index was below five stars, in line with previously published quality-control criteria [27], [28], [29].

All examinations were performed with participants in a standardized supine position with head support. Ample ultrasound gel and minimal probe pressure were used to avoid tissue precompression. For assessment of the sternocleidomastoid muscle, the transducer was aligned longitudinally to the muscle fibers and positioned at an oblique angle of approximately 45°, ensuring a scanning plane perpendicular to the muscle bulk while accounting for muscle anisotropy [31], [32].

All SWE measurements were performed by a single experienced operator with extensive expertise in musculoskeletal ultrasound and shear-wave elastography, routinely performing such examinations since 2011 (approximately 100–200 examinations per week). Although no formal intra- or interobserver reproducibility analysis was conducted, SWE fundamentally differs from strain elastography in that tissue excitation is generated by system-controlled acoustic radiation force rather than manual compression, and previous Resona-7-based studies have demonstrated good to excellent reproducibility under standardized conditions [28], [29], [30], [31], [33], [34].

SWE primarily measures shear-wave propagation velocity, from which Young’s modulus was derived assuming a constant soft-tissue density of 1000 kg/m^3^. Stiffness values were therefore reported in kilopascals (kPa), consistent with established practice in musculoskeletal SWE literature [31], [32].

Statistical analysis was conducted using the Pandas data analysis toolkit. To compare groups, paired and unpaired t-tests were employed for parametric distributions, while the Mann-Whitney U Test was used for non-parametric distributions. Correlation analyses were performed with the Spearman rank correlation coefficient for non-parametric distributions and the Pearson correlation coefficient for parametric distributions. Details of the power analysis (post-irradiated vs. controls) can be found in the supplementary material A.

## 3 Results

**Fig. 1:**
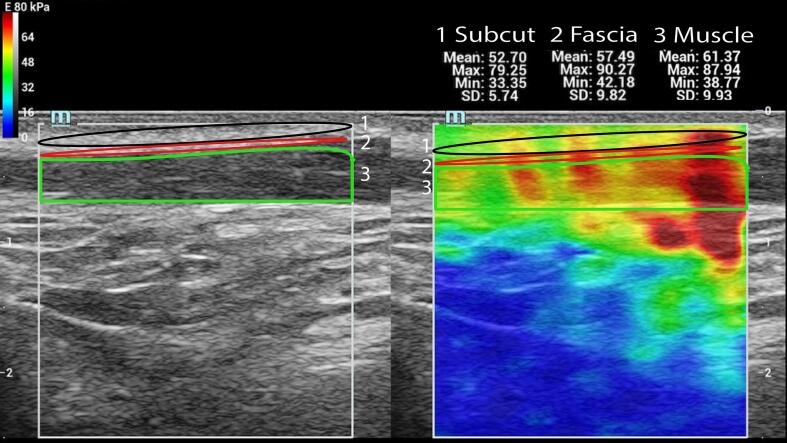
Shear-wave elastography of the sternocleidomastoid muscle: 1 Trace of the subcutaneous tissue (Subcut) with EMean of 52.70 kPa (black frame), 2 Trace of the fascia with EMean of 57.49 kPa (red frame), 3 Trace of the muscle (green frame) with an EMean of 61.37 kPa. (For interpretation of the references to colour in this figure legend, the reader is referred to the web version of this article.)

Stiffness was significantly higher in the OG than in the CG across muscle, fascia, and subcutaneous tissue for all mean, maximum, and minimum measurements (Table 2). The largest differences were observed in muscle, with approximately twofold higher stiffness in the OG compared with the CG (right side: 46.75 vs. 21.52 kPa, p < 0.001). Similar increases were found in the fascia, whereas differences in subcutaneous tissue were less pronounced. An example SWE acquisition is shown in Fig. 1.

**Table 2:** Stiffness in kPa of the muscle, fascia, and subcutaneous tissue.

**Measurement**	**OG Med**	**CG Med**	**Ratio**	**OG MAD**	**CG MAD**	**p-value**
Muscle Mean L	46.98	28.10	1.67	22.29	7.14	0.001
Muscle Mean R	46.75	21.52	2.17	16.51	9.55	< 0.001
Fascia Mean L	46.41	25.47	1.82	21.80	8.97	0.002
Fascia Mean R	44.28	22.69	1.95	21.61	8.95	0.001
Subcutaneous Mean L	34.16	23.89	1.43	12.60	6.72	0.001
Subcutaneous Mean R	34.31	20.92	1.64	10.16	7.05	0.001
Muscle Max L	99.87	59.04	1.69	38.60	25.24	0.001
Muscle Max R	98.88	47.72	2.07	49.99	23.74	< 0.001
Fascia Max L	67.56	47.35	1.43	31.41	16.68	0.003
Fascia Max R	74.24	34.68	2.14	43.52	17.48	0.006
Subcutaneous Max L	48.57	41.46	1.17	18.17	10.29	0.006
Subcutaneous Max R	47.54	37.97	1.25	16.88	14.36	0.015
Muscle Min L	19.27	12.88	1.50	9.41	4.28	0.014
Muscle Min R	20.10	10.06	2.00	7.52	3.40	< 0.001
Fascia Min L	26.94	14.57	1.85	11.76	3.60	0.001
Fascia Min R	27.31	14.47	1.89	9.35	3.83	0.001
Subcutaneous Min L	24.68	13.34	1.85	9.51	5.68	< 0.001
Subcutaneous Min R	25.10	14.13	1.78	9.41	5.42	0.001

OG = observation group, Med = median, CG = control group, MAD = median absolute deviation, L = left, R = right.

Muscle and skin thickness were significantly reduced in the OG compared with the CG, whereas subcutaneous tissue thickness did not differ significantly between groups (Table 3).

**Table 3:** Tissue thickness in millimeters (mm).

**Region**	**OG Mean**	**CG Mean**	**SD**		**p-value**
Muscle L	7.2	9.8	±2.65		< 0.001
Muscle R	7.8	10.3	±2.27		< 0.001
Subcutaneous L	1.5	1.5	±0.69		0.900
Subcutaneous R	1.3	1.7	±0.71		0.090
Skin L	1.5	1.9	±0.38		< 0.001
Skin R	1.5	2.0	±0.45		< 0.001

OG = observation group, CG = control group, SD = standard deviation, L = left, R = right.

There were small differences in neck rotation between the two groups, including approximately 5° lower cervical rotation in the OG; however, these differences were not statistically significant. There were also no differences in flexion, extension, or lateral flexion (Table 4).

**Table 4:** Cervical range of motion.

**Measure**	OG Mean*/Median**	**CG** Mean*/Median**	**p-value**
Rotation L	52.4*	57.4*	0.295
Rotation R	53.7*	58.6*	0.262
Flexion	57.1*	51.8*	0.153
Extension	45.5*	43.7*	0.721
Lateral Flexion L	26.0**	26.0**	0.855
Lateral Flexion R	24.5**	24.5**	0.545

Cervical range of motion is given in degrees (°); OG = observation group, CG = control group, r = Pearson r, L = left, R = right.

In both the OG and CG, moderate negative correlations were observed between muscle stiffness and cervical range of motion (CROM); however, only left lateral flexion in the CG reached statistical significance (r = -0.53, p = 0.034) (Table 5).

**Table 5:** Correlation between muscle stiffness and cervical range of motion.

**Parameter**	**OG** **r**	**OG** **p-value**	**CG** **r**	**CG** **p-value**
Rotation L	−0.27	0.318	−0.41	0.117
Rotation R	−0.20	0.468	−0.46	0.072
Flexion	−0.31	0.248	−0.17	0.536
Extension	−0.41	0.115	−0.25	0.356
Lateral Flexion L	−0.36	0.167	−0.53	0.034
Lateral Flexion R	−0.13	0.621	−0.46	0.070

Muscle stiffness values represent mean bilateral muscle stiffness (kPa) derived from shear wave elastography measurements. Pearson correlation coefficients are reported. CG = control group; OG = observation group; L = left; R = right.

In both groups, muscle stiffness showed consistently negative correlations with cervical range of motion, indicating reduced mobility with increasing stiffness. However, these associations were generally weak to moderate and did not reach statistical significance in the observation group. In the control group, a moderate negative correlation was observed for left lateral flexion (r =  − 0.53, p = 0.034), while all other correlations remained non-significant.

Correlations between stiffness metrics and EORTC QLQ-C30 global health as well as EORTC QLQ-HN35 symptom scales were weak (range –0.322 to 0.266), and associations with lymphedema were negligible (range –0.256 to 0.024).

## 4 Discussion

To our knowledge, this is the first study to quantify radiation-induced cervical fibrosis in terms of stiffness in a tissue-specific manner using SWE. There was significant hardening of all tissue types compared with controls, with an approximately twofold increase in stiffness of muscle and fascia, whereas changes were less pronounced in subcutaneous fatty tissue. The values can be regarded as reliable due to standardized acquisition and consistent bilateral measurements, underlining the suitability of SWE for quantifying radiation-induced cervical fibrosis. The observed stiffness increase represents a clinically meaningful effect size, even in the presence of largely preserved CROM. While inverse associations between muscle stiffness and cervical range of motion were observed, these did not reach statistical significance in the OG. In contrast, moderate negative correlations were more evident in the CG. This finding suggests that the relationship between tissue stiffness and functional mobility may be altered following radiotherapy. Possible explanations include compensatory mechanisms, mild symptom burden, and limited statistical power due to small sample size. Together, these findings suggest a dissociation between structural tissue alterations and functional impairment in irradiated patients.

Such subclinical tissue stiffening may indicate reduced tissue compliance and adaptability, even in the absence of measurable functional impairment. These findings highlight the clinical value of SWE in detecting subclinical or compensated stages of radiation-induced fibrosis not captured by routine assessment.

In a previous study by Liu et al., which also investigated muscle stiffness in ENT cancer patients after radiotherapy, the stiffness values (E) had a normal distribution, whereas in this study, only subcutaneous tissue of the right sternocleidomastoid muscle demonstrated normal distribution [14]. Reported stiffness ratios in that study were higher than those observed here, while dispersion was also greater, as reflected by higher variability compared with MAD-adjusted measures. These differences are likely attributable to the larger ROI size used in our study, resulting in more homogeneous measurements.

Radiation primarily harms tissues by triggering apoptosis or clonogenic cell death through DNA damage caused by free radicals, resulting in inflammatory responses [35]. Increased expression of mediators such as connective tissue growth factor and TGF-β promotes myofibroblast activation and collagen and fibronectin deposition, leading to progressive tissue stiffening [13], [36], [37], [38], [39]. Despite these established mechanisms, the full pathophysiological complexity of radiation-induced fibrosis remains incompletely understood, including potential effects on peripheral nerve function, which may arise from fibrotic compression, ischemia, or both [9]. In the present study, stiffening effects were more pronounced in muscle and fascia than in subcutaneous tissue, which may reflect increased myofibroblast activity with greater collagen incorporation in these compartments. This finding is consistent with emerging concepts of fascia as a dynamic, multi-layered, mechanosensitive tissue system rather than a passive supportive structure [40], [41], [42]. In addition, a not-yet-fully-understood tissue “densification” may contribute, supported by the observed reduction in muscle and fascia thickness compared with controls, in contrast to subcutaneous tissue [43].

Dose–volume relationships in radiation-induced fibrosis have been demonstrated previously [40], and muscle dose has been shown to correlate with functional impairment, such as trismus after irradiation of the pterygoid muscles [44], [45]. The homogeneous increase in tissue stiffness and moderate clinical symptoms observed in our cohort are likely explained by a uniform dose prescription (50 Gy) and strict plan quality using intensity modulated radiotherapy [12]. The good preservation of cervical spine mobility may further reflect effective myelon sparing and lower doses to cervical vertebral joints enabled by volumetric arc therapy, with largely preserved muscle function despite increased tissue density. Consequently, the lack of significant correlations between stiffness and functional parameters may reflect preserved functional compensation despite measurable structural alterations.

These findings suggest that radiation-induced tissue changes may not directly translate into measurable functional impairment, particularly in early or moderate stages. Owing to the lack of a unified definition of radiation-induced fibrosis [1], and limited dose–effect correlations for soft tissue complications, further clarification of pathophysiological mechanisms and clinically relevant endpoints is required. In this context, tissue-specific and reproducible fibrosis quantification by SWE offers strong potential for future dose–volume and normal tissue complication probability analyses in larger cohorts.

One limitation of this study was the small sample size of the OG, combined with recruitment during routine follow-up visits, which may have introduced positive selection bias toward patients with good recovery and contributed to the generally mild long-term effects observed. The limited cohort size also restricted the detection of robust correlations between tissue stiffness and CROM as well as QoL, particularly given substantial interindividual variability. Another limitation is the lack of formal intra- and interobserver reproducibility analyses for SWE measurements. Although examinations were performed by a single experienced operator using standardized protocols, quantitative reliability metrics were not assessed.

In conclusion, cervical fibrosis after radiotherapy predominantly affects muscle tissue and associated fascia, with stiffness values approximately twice as high as in the general population. Despite these structural changes, functional impairment remains limited and is not directly reflected by significant correlations with cervical range of motion. SWE represents a promising tool for reliable, tissue-specific quantification of radiation-induced fibrosis and may support future efforts to better define dose constraints and targeted rehabilitation strategies.

## Ethical approval declarations

The study was approved by the Ethics Committee of the Technical University of Munich and conducted in accordance with the institution’s ethical standards (ethical vote: 360/Sarva S-EB).

## Declaration of generative AI and AI-assisted technologies in the writing process

During the preparation of this work, the author(s) used ChatGPT (OpenAI) in order to support language editing and improve grammar, clarity, and readability of the manuscript text. After using this tool, the authors reviewed and edited the content as needed and take full responsibility for the content of the published article.

## CRediT authorship contribution statement

**Hendrik Dapper:** Writing – review & editing, Writing – original draft, Visualization, Validation, Supervision, Software, Project administration, Methodology, Investigation, Formal analysis, Data curation, Conceptualization. **Maria Waltenberger:** Writing – review & editing, Validation, Methodology, Investigation. **Steffi U. Pigorsch:** Writing – review & editing, Validation, Resources, Methodology, Conceptualization. **Stephanie E. Combs:** Writing – review & editing, Validation, Supervision, Resources. **Katharina Bauermeister:** Writing – review & editing, Validation, Software, Resources, Investigation, Data curation. **Wolfgang Bauermeister:** Writing – original draft, Visualization, Validation, Software, Resources, Methodology, Investigation, Formal analysis, Data curation.

## Informed consent

Informed consent was obtained from all subjects and/or their legal guardian(s).

## Funding

This work has not received any special grants.

## Declaration of competing interest

The authors declare that they have no known competing financial interests or personal relationships that could have appeared to influence the work reported in this paper.

## Data Availability

Most data generated or analyzed during this study are included in this published article. Further datasets are available from the corresponding author on reasonable request.

## Appendix A Supplementary data

Supplementary Data 1 Additional Methodological Details.
