# Supplementary material for: Shear-wave ultrasound elastography for tissue-specific quantification of radiation-induced cervical fibrosis
Source: Phys Imaging Radiat Oncol. 2026 May 27;39:101005. doi: 10.1016/j.phro.2026.101005 (PMC13241838; doi:10.1016/j.phro.2026.101005)
Supplement: Supplementary Data 1 — Additional Methodological Details. [file mmc1.docx]

**Supplementary Material**

Shear-Wave Ultrasound Elastography for Tissue-Specific Quantification of Radiation-Induced Cervical Fibrosis

**Supplementary material A**


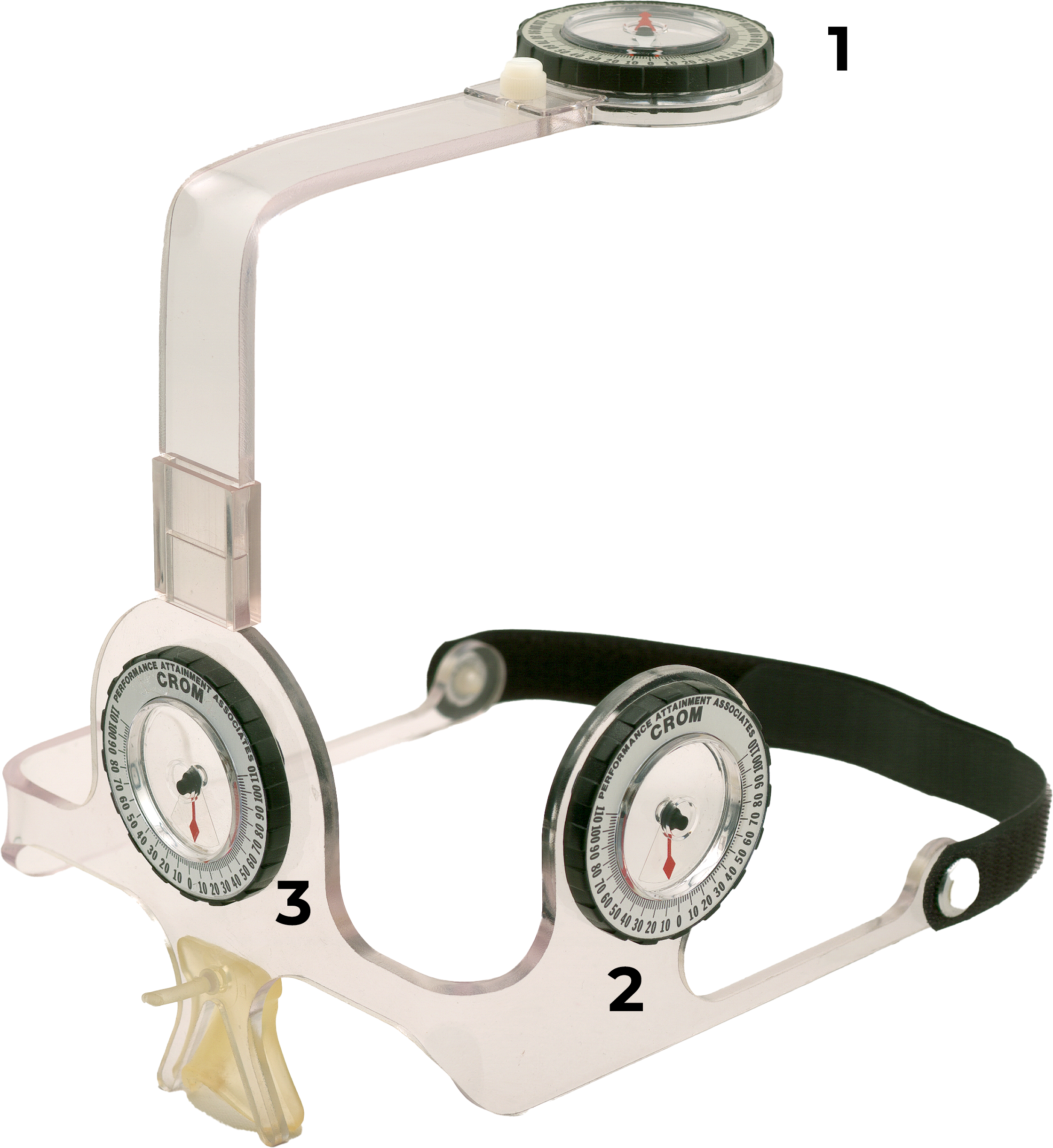


**Figure S1** Cervical Range of Motion Device (CROM) 1 Compass goniometer for rotation measure- ment, 2 Gravity goniometer for flexion and extension measurement, 3 Gravity goniometer for lateral flexion measurement


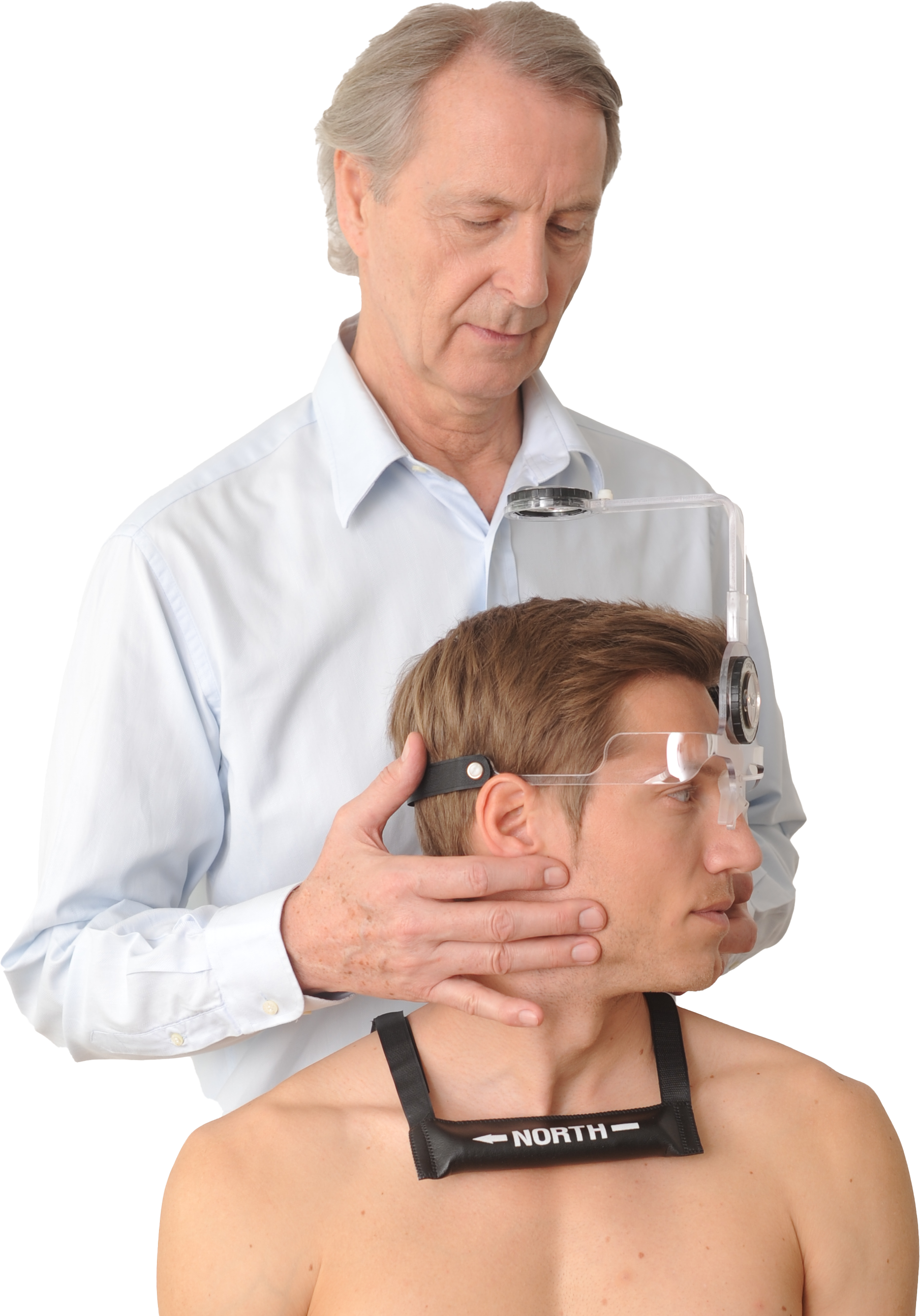


**Figure S2** Rotation measurement with the compass goniometer of the CROM device. A magnetic yoke stabilizes the compass needle, compensating for the influence of electromagnetic fields.


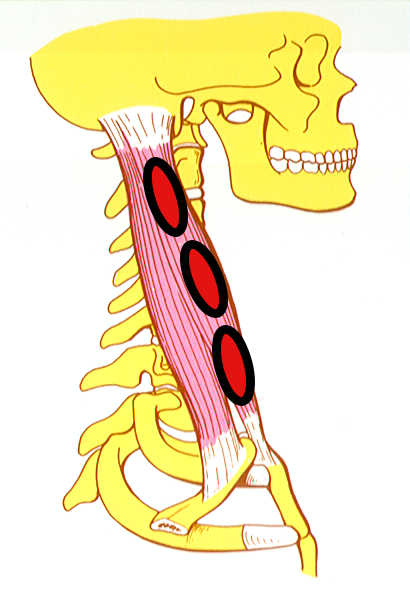


**Figure S3** Schematic illustration of the positioning of the ultrasound probe along the sternocleidomastoid muscle.

**Power Analysis** **(Post-irradiated vs. Controls)**

**Method of Power Analysis**

Comparison: Independent groups (post-irradiated patients vs. controls) for each measurement site.

Effect size: Cohen’s d = (M_post − M_control) / SD_pooled, where SD_pooled = sqrt((SD_post² + SD_control²) / 2).

Test: Two-sided t-test, α = 0.05, 1:1 allocation.

Assumed sample size for power estimation: n = 16 per group.

Data source: Means and standard deviations provided.

**Main Results (n = 16 per group)**

All measurement sites showed markedly higher stiffness in the post-irradiated group. Absolute differences ranged from +39.6 to +72.8 kPa. Standardized effect sizes were consistently large (d = 1.01–1.35). The corresponding statistical power was high—≥ 0.85 for all but one site (range 0.79–0.96). Reported p-values (<0.001) are consistent with these large effects.

**Site Δ Mean (kPa) Cohen’s d Power (n=16)**

L mid SCM 47.7 1.15 0.88

L low SCM 44.3 1.35 0.96

R mid SCM 48.8 1.14 0.88

R low SCM 56.1 1.16 0.89

Mean SCM 44.7 1.33 0.95

L mid superficial fascia 39.6 1.11 0.86

L low superficial fascia 51.1 1.18 0.90

R mid superficial fascia 42.9 1.01 0.79

R low superficial fascia 72.8 1.09 0.85

Mean superficial fascia 48.6 1.28 0.94

**Highlights**

• Largest standardized effect: L low SCM (d = 1.35; Power 0.96)

• Largest absolute difference: R low superficial fascia (+72.8 kPa; d = 1.09; Power 0.85)

• Compartment means: Mean SCM d = 1.33 (Power 0.95), Mean superficial fascia d = 1.28 (Power 0.94)

• Only one site fell slightly below the conventional 0.80 threshold for adequate power: R mid superficial fascia (Power 0.79)

**Sample Size Planning (Future Studies)**

Required n per group for 80% power (α = 0.05, two-sided), based on observed d-values:

• 10–11 per group are sufficient for Mean SCM (d = 1.33) and Mean superficial fascia (d = 1.28)

• 10–15 per group for most individual sites (e.g., L mid SCM 13; R low superficial fascia 15)

• Up to 17 per group if aiming to detect the smallest observed effect (R mid superficial fascia, d = 1.01) with ≥0.80 power individually

**Interpretation**

The post-irradiated group exhibited markedly increased tissue stiffness of both the sternocleidomastoid (SCM) and the superficial fascia compared to controls. The large effect sizes indicate clinically relevant differences, and with n = 16 per group, statistical power was high for almost all sites. For future studies, n ≈ 12–15 per group will generally be sufficient; only the weakest effect would require ≈ 17 per group for robust detection.

Note: This power analysis is based on group means/SDs and the assumption of independent samples with a two-sided t-test. If variances differ substantially or distributions deviate from normality, robust or transformed analyses should be considered in addition.
